# Sources of variability in cytosolic calcium transients triggered by stimulation of homogeneous uro-epithelial cell monolayers

**DOI:** 10.1098/rsif.2014.1403

**Published:** 2015-04-06

**Authors:** Peter A. Appleby, Saqib Shabir, Jennifer Southgate, Dawn Walker

**Affiliations:** 1 Department of Computer Science/INSIGNEO Institute for *In Silico* Medicine, University of Sheffield, Sheffield, UK; 2 Jack Birch Unit for Molecular Carcinogenesis, Department of Biology, University of York, York, UK

**Keywords:** heterogeneity, automated image analysis, calcium signalling, urothelium, epithelium, wound repair

## Abstract

Epithelial tissue structure is the emergent outcome of the interactions between large numbers of individual cells. Experimental cell biology offers an important tool to unravel these complex interactions, but current methods of analysis tend to be limited to mean field approaches or representation by selected subsets of cells. This may result in bias towards cells that respond in a particular way and/or neglect local, context-specific cell responses. Here, an automated algorithm was applied to examine in detail the individual calcium transients evoked in genetically homogeneous, but asynchronous populations of cultured non-immortalized normal human urothelial cells when subjected to either the global application of an external agonist or a localized scratch wound. The recorded calcium transients were classified automatically according to a set of defined metrics and distinct sub-populations of cells that responded in qualitatively different ways were observed. The nature of this variability in the homogeneous cell population was apportioned to two sources: intrinsic variation in individual cell responses and extrinsic variability due to context-specific factors of the environment, such as spatial heterogeneity. Statistically significant variation in the features of the calcium transients evoked by scratch wounding according to proximity to the wound edge was identified. The manifestation of distinct sub-populations of cells is considered central to the coordination of population-level response resulting in wound closure.

## 1. Introduction

Epithelia are tissues that line the cavities and surfaces of the body where they form the interface with the external environment. Maintaining the integrity of these barriers is an essential function of epithelial tissue homeostasis. An example is the urinary barrier found lining the lumen of the bladder and associated urinary tract, which comprised a specialized epithelium known as urothelium. Damage to the urothelium triggers a rapid, regenerative response leading to self-repair and restitution of barrier integrity. This involves a range of responses within the population of cells that make up the urothelium with, for example, cells entering migratory and/or proliferative modes. Urothelial wound repair can be investigated experimentally using a normal human urothelial cell culture system, where studies involving scratch wounding of confluent monolayers have been useful in identifying key signalling pathways [1–4]. Agent-based and multi-scale computational modelling of cell populations can provide a useful adjunct to experimental cell biology for testing understanding [5,6] (and reviewed in [7]), with iterative cycles of experiment and modelling able to reveal new biological insight [8].

A critical question that arises is how the initiation of individual cell responses results in cooperative wound-closing behaviour at the population level. We and others have proposed that the triggering, transmission and individual response to calcium signalling is responsible for the localized coordination of cell response within the population, both *in vitro* [3,9,10] and *in vivo* [11,12] (reviewed in [13]). Here, we have explored the association between the nature of the extrinsic signal and the resulting calcium transient or ‘signature’ triggered within each individual cell. Our approach is driven by the hypothesis that the context-specific calcium signature plays a key role in coordinating population-level behaviour by defining the individual cell response. Towards this end, we have recently developed a framework for modelling calcium transients in specific cell types, using urothelium as our exemplar [14].

In order to understand how a calcium signature is translated into a behavioural response, it is important to understand how different calcium transients can arise in individual cells within a homogeneous population. Our first aim was, therefore, to characterize both the degree and the origin of variability of calcium signatures in a genetically homogeneous cell population. The issue of variability is often overlooked in the experimental literature, where cell populations are either characterized using mean-field measurements, or by examining a small sample of individual cell responses. Although in the right context these approaches can be appropriate, their use rests on implicit assumptions of uniformity and the validity of these assumptions is not well understood.

Here, we studied the heterogeneity in calcium responses generated within an adherent confluent monolayer of cultured normal human urothelial cells by examining factors contributing to the variability in response between individual cells in the population. Firstly, we examined what we refer to here as the *intrinsic variability* in individual cell response. For this, we applied an experimental protocol where the adherent cell monolayer was perfused with medium containing adenosine triphosphate (ATP), which is a purinergic agonist that is the principal extracellular signalling factor released during wounding of urothelial cells and has been shown to induce calcium transients when applied as an exogenous factor [3,15]. To avoid any bias implicit in the analysis of a selected sub-population of responding cells, an automated technique to segment a majority of cells from a time-lapse series of immunofluorescence images was developed, based on the watershedding technique [16,17]. Once these regions of interest (ROIs) were identified, the individual cell calcium transients were obtained by extracting pixel intensities associated with the ROIs for all images in the time series. Owing to the large quantity of data generated by this approach, an automated computer vision/image analysis algorithm was applied that identified and classified the cell responses according to key features of the calcium signature. Scratch-wounding experiments were also carried out to uncover a second determinant of an individual cell's response in the form of the context-specific dependence on the spatial location of a cell relative to the wound site. This provides an extrinsic source of variability and in our discussion, we have considered the combination of intrinsic and extrinsic variability in terms of the emergence of population-level responses such as wound closure, as well as wider implications for the analysis and interpretation of cell imaging data in general.

## 2. Results

### 2.1. Induction and automated extraction of calcium transients in urothelial cell cultures

**Figure 1.**
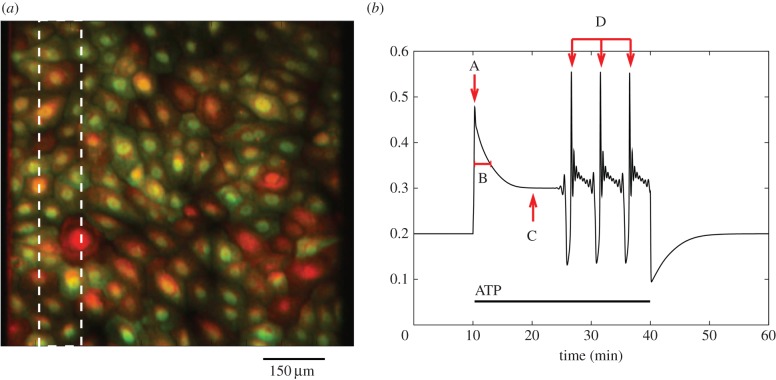
To illustrate a cultured urothelial cell monolayer and key characteristics of the measured calcium transients used in the transient classification algorithm. (*a*) Example of a cultured urothelial cell monolayer. Cultures were first grown to form a confluent monolayer then loaded with the calcium-binding fluo4 and fura red dyes, which, respectively, reduce and increase in intensity of fluorescence emission when bound to calcium and can be used ratiometrically to correct for loading inequalities between cells. Cultures were stimulated by either perfusing ATP over the cell layer or removing a strip of cells using a glass pipette (white dotted box). (*b*) Key characteristics used in the calcium transient classification algorithm. After some pre-processing steps to reduce the amount of noise in the trace, the following information was recorded: (A) the maximum of the initial peak, (B) the time taken for the calcium to fall to half this initial peak value (the full-width half-maximum, or FWHM), (C) the magnitude of the plateau and (D) the calcium spike interval (if the cell subsequently enters a spiking ode). This set of features provides sufficient information to classify the population of transients into several distinct groups.

Changes in cytosolic calcium concentration in a confluent monolayer of cultured urothelial cells were visualized using epifluorescent microscopy and recorded digitally over time, as illustrated in figure 1*a*. Cells preloaded with calcium-binding fluorescent dyes were stimulated either by application of the purinergic agonist ATP in a laminar flow perfusion chamber or by wounding of the cell layer using a sharpened implement to remove a strip of cells. Full details of these two experimental protocols are given in the Experimental methods section. It should be noted that both ATP perfusion and wounding experiments were repeated a total of three times on independent donor cell lines. Figures show results from one experiment only for reasons of clarity; results from replicates are included in tables 1 and 5.

**Table 1.** Classification of calcium transients evoked using the ATP protocol.

		number of cells				
		replicate				
primary class	secondary class	1 (*n* = 114)	2 (*n* = 117)	3 (*n* = 126)	per cent of population (mean ± s.e.m.)	figure
(1) slow or absent response	(1a) non-responding	12	11	8	21 ± 3%	4*a*
	(1b) slowly rising	8	13	11		4*b*
	(1c) slowly rising–peak–baseline	4	5	1		4*c*
(2) rapid peak, then plateau	(2a) peak–plateau	27	24	31	33 ± 1%	4*d*
	(2b) peak–plateau–peak–plateau	5	3	6		4*e*
	(2c) peak–baseline–spiking–plateau	7	10	3		4*f*
(3) rapid peak, then spiking	(3a) peak–baseline–peak–baseline	6	3	4	47 ± 3%	4*g*
	(3b) peak–baseline–spiking	24	29	33		4*h*
	(3c) peak–plateau–spiking	21	19	29		4*i*

**Figure 2.**
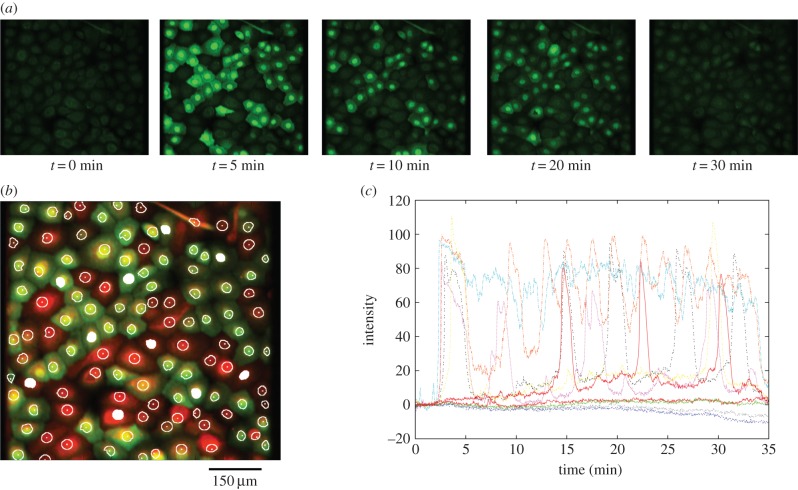
Calcium transients triggered by the application of the purinergic agonist ATP to the urothelial cell monolayer. (*a*) Time-lapse fluorescence microscopy images of the urothelial cell monolayer using a ×20 objective. (*b*) Segmentation of cells to identify 114 ROIs. (*c*) Calcium transients obtained from 10 representative cells after segmentation of the image using the ROI algorithm (indicated by the white-filled ROIs in *b*). These were selected to illustrate the variability in the responses observed, including bursting, spiking and extended plateaus. Colours are arbitrary and for visual clarity. Intensity is in arbitrary units.

Exogenous application of ATP induced an elevation in cytosolic calcium concentration in cultured urothelial cells. The results were captured as a time-lapse sequence of epifluorescence microscopy images, with changes in fluorescence intensity indicating changes in cytosolic calcium concentration, as illustrated in figure 2*a*. An automated segmentation or ROI extraction algorithm (described in §4.2) was applied to automatically delineate a maximum number of individual cells, thus eliminating any bias over selection. Figure 2*b* shows the result of applying this algorithm to the sequence of images produced using the ATP stimulation protocol. In order to optimize and maximize individual cell identification, the reciprocal red and green images produced by the two fluorescent dyes (see Experimental methods) at the beginning and end of the time-lapse sequence were combined to create a single red–green image. In total, 114 individual ROIs are identified from this image, which corresponded to 96% of the total population (as counted by hand from the number of nuclei).

### 2.2. Classification of calcium transients

An examination of the time-lapse movies for the 114 ROIs revealed a broad range of responses ranging from bursting and spiking behaviours, to pronounced plateaus, to cells that responded minimally to exogenous ATP. The spectrum of observed behaviours is illustrated in the range of transients shown in figure 2*c*. The population of urothelial cells, despite being apparently homogeneous and experiencing an identical external stimulus, therefore, exhibited intrinsic variability in their individual responses.

We next introduced a classification scheme to analyse the calcium transients based on the following features: the magnitude of the initial peak, the time taken for the calcium transient to fall to a half of the initial peak (the full-width half-maximum, or FWHM), the magnitude of the subsequent plateau, and the rate of spiking or bursting that followed. An illustration of this classification algorithm is shown in figure 1*b*.

**Figure 3.**
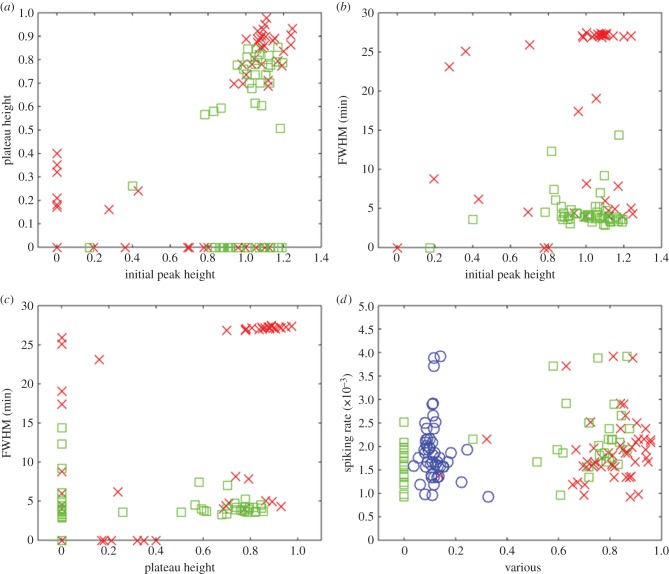
Detailed analysis of the calcium transients recorded during exposure of cells to exogenous ATP. (*a*) Initial peak height versus plateau height for cells (both in arbitrary units) which subsequently did not enter a spiking mode (red, ×) and those which did (green, □). Three clusters were identified. Small initial peaks only produced shallow plateaus. A larger initial peak can lead to one of two behaviours: a shallow plateau followed by spiking, or a higher plateau that tends to be stable. (*b*) Initial peak height (in arbitrary units) versus FWHM (which indicates the plateau duration) for the same population of cells. Again, we can identify three separate clusters. Small initial peaks produce short plateaus. A larger initial peak leads to two clearly separated groups: those with an extended plateau that do not subsequently start spiking, and those with a short plateau that subsequently give way to spiking. (*c*) Plateau height (in arbitrary units) versus FWHM. Contrast non-spiking cells, which tend to have a long, high plateaus compared to spiking cells, which have a continuous spread of plateau heights but of much shorter duration. (*d*) Initial peak height (red, ×), plateau height (green, □), and FWHM (blue, ○) versus spiking rate (in s^−1^), for cells which subsequently entered a spiking or bursting mode. There is no obvious relationship between any of these features and the subsequent spiking or bursting rate. Note that in each case the abscissa has been scaled so that the data points lie between 0 and 1.

Figure 3 contains four plots that illustrate the relationship between the magnitude of the initial peak, the FWHM, the magnitude of the subsequent plateau and the rate of any spiking or bursting that follows. We made a distinction between cells that subsequently entered a spiking mode from those that did not, as these responses represent qualitatively distinct modes of behaviour. Figure 3*a* shows the initial peak height versus plateau height, from which three clusters of points can be identified. The first, very sparse cluster in the bottom left region indicates that small initial peaks in calcium concentration only ever produced a very shallow plateau. This group, which accounted for approximately 10% of the data points, corresponded to cells that responded very little to the ATP stimulation. A larger initial peak was observed to lead to one of several behaviours. The bottom right cluster represents cells that showed a large initial peak followed by a return to baseline, a behaviour that was typically followed by spiking or bursting. The top right cluster represents cells that had a large initial peak followed by a high plateau that was sometimes stable and sometimes gave way to spiking.

Figure 3*b* shows the initial peak height versus FWHM for the same population of cells. In this context, the FWHM indicates the duration of the plateau that followed the initial peak. The cluster of points in the bottom left of figure 3*a* is preserved in this plot but, as the transients that comprised it did not have an initial peak, the FWHM is also, by definition, zero, meaning the points are coincident. For transients with a higher initial peak, we observed two main clusters of points. The top right cluster indicates that peaks that were slow to decay always led to a stable plateau. The bottom right cluster indicates that peaks that fell away more rapidly tended to give way subsequently to spiking.

Figure 3*c* shows the plateau height versus the FWHM. There are three distinct clusters in this plot. The top right cluster indicates that non-spiking cells tend to have a long, high plateau. The cluster in the bottom left is composed of cells that lack a plateau phase and which subsequently entered a spiking mode. The cluster in the bottom right indicates cells that had a high, but fairly short plateau, mostly followed by spiking. Figure 3*d* shows the initial peak height, plateau height and FWHM versus the spiking rate, for cells that subsequently entered a spiking mode. The clusters observed in the other plots are preserved as groups of points along the horizontal axis, but each group exhibited a continuous distribution of spiking rates extending up the vertical axis, indicating that there was very little correlation between features associated with the transient and the spiking rate that followed. In other words, for cells that start to spike we cannot reliably predict the eventual rate of spiking by inspecting the calcium transient that preceded it.

From examining figure 3*a*–*c*, a number of distinct groups that correspond to different classes of calcium transient can be identified. An important distinction was between cells that responded immediately to the application of ATP and subsequently entered a plateau or spiking mode, and those that exhibited a delayed or non-measurable response. These are designated as the primary transient categories listed in tables 1–3. These three classes are approximately equally represented within the cell ensemble, and together they describe the entire population of 114 cells. Each of these primary categories can be subdivided into three secondary groups as described below (see also tables 1–3). The clusters observed in figure 3 can be understood in terms of the nine distinct classes of transient.

**Figure 4.**
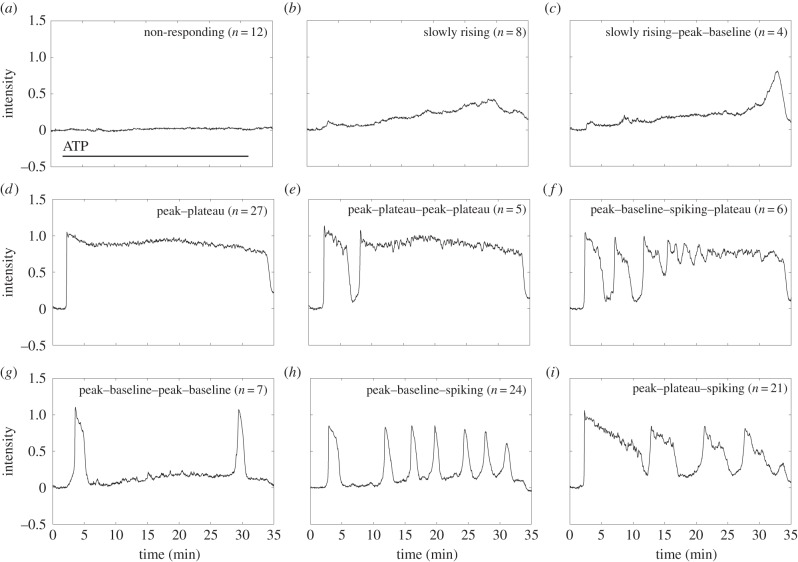
Examples of each of the nine classes of calcium transient observed when cells were exposed to 25 μM ATP for 35 min. (*a–c*) Transients that were slowly responding in nature; (*d*–*f*) transients that were rapidly responding and led to a stable plateau mode and (*g*–*i*) transients that were rapidly responding and had a long-term behaviour characterized by one or more rapid elevations followed by a return to baseline. All intensities are in arbitrary units. Together these nine classes of transient describe the entire population of 114 identified cells in the field of view.

Examples of cells that had a delayed or non-measurable response are shown in figure 4*a–c*. These are subdivided into three qualitatively distinct kinds of transient, examples of which are shown in figure 4*a–c*: (i) non-responding (*n* = 12, figure 4*a*); (ii) slowly rising (*n* = 8, figure 4*b*) and (iii) slowly rising followed by a peak and return to baseline (*n* = 4, figure 4*c*). Together these account for 21% of the total population.

Cells that responded quickly to the application of ATP (figure 4*d–i*) were more varied than the slowly responding group and formed two subsets. The first included transients that responded with an initial peak that transitioned to: (i) a long, high plateau (*n* = 27 figure 4*d*); (ii) a short plateau followed by a second peak–plateau (*n* = 5, figure 4*e*) and (iii) a return to baseline then entry into a transient mode (*n* = 6, figure 4*f*).

The characteristic feature of these three classes of transient is that they all end in an extended plateau, and together these account for 34% of the population.

Transients within the second subset of responsive cells were characterized by a long-term behaviour dominated by rapid elevations in cytosolic calcium concentration, followed by a return to baseline. These are: (i) a double peak–baseline, (*n* = 7, figure 4*g*); (ii) a peak–baseline followed by spiking, (*n* = 24, figure 4*h*) and (iii) a peak, followed by a plateau, then spiking, (*n* = 21, figure 4*i*). Together these account for 45% of the population.

Table 1 also includes the breakdown of the number of transient types observed in all three replicates, and it can be seen that there is close agreement between the three experiments generated using independent cell lines as biological replicates. Table 2 contains the mean and s.e.m. for the different transient characteristics, and table 3 contains the results of statistical significance testing for various signal characteristics according to the primary classifications of cytoplasmic calcium response. As expected, table 2 indicates that cells identified as belonging to the slow/non-responding group had a smaller maximum peak amplitude and longer response time than cells in other groups, whereas cells whose response characteristic was dominated by an extended plateau exhibited a larger plateau height and response duration (FWHM). It can be seen that each primary transient type had several characteristics that were significantly different to those obtained in other groups, thus suggesting that our method of classification is valid.

**Table 2.** Mean and s.e.m. for various signal characteristics according to the primary classifications of cytoplasmic calcium response identified after the application of ATP. Peak and plateau heights are in arbitrary units.

	mean ± s.e.m.				
primary class	peak *h*	peak *t* (min)	FWHM (min)	plateau height	spiking rate (s^−1^)
(1) slow or absent response	0.11 ± 0.05	6.95 ± 3.26	0.72 ± 0.61	0.07 ± 0.03	0
(2) rapid peak, then plateau	1.04 ± 0.03	1.13 ± 0.01	3.92 ± 0.72	0.79 ± 0.03	(1.1 ± 0.6) × 10^−3^
(3) rapid peak, then spiking	0.98 ± 0.03	4.20 ± 0.92	1.81 ± 0.11	0.28 ± 0.05	(1.9 ± 0.4) × 10^−3^

**Table 3.** Statistical significance testing for different signal characteristics according to the primary classifications of cytoplasmic calcium response identified after the application of ATP. The three primary signal classes were compared using the non-parametric Kruskal–Wallis test with Dunn's multiple comparisons post-test. Results indicating a statistical difference in the sample medians are indicated with asterisks.

		*p*-value				
primary class	comparison group	peak *h*	peak *t*	FWHM	plateau height	spiking rate
(1) slow or absent response	(2) rapid peak, then plateau	***<0.001	>0.05	***p < 0.001	***<0.001	>0.05
	(3) rapid peak, then spiking	***<0.001	***<0.001	***p < 0.001	>0.05	***<0.001
(2) rapid peak, then plateau	(3) rapid peak, then spiking	>0.05	***<0.001	>0.05	***<0.001	***<0.001

### 2.3. Spatial variability in calcium transients following wounding

In order to consider if the heterogeneity in calcium response was inherent to the population or could be modified by local (extrinsic) factors, we examined the calcium transients recorded during a scratch-wounding experiment where a strip of cells was physically removed from the urothelial cell monolayer using a sharp implement. The mechanical stimulus/injury induced by this process has been shown previously to induce a calcium transient in a wounded cell monolayer [3].

**Figure 5.**
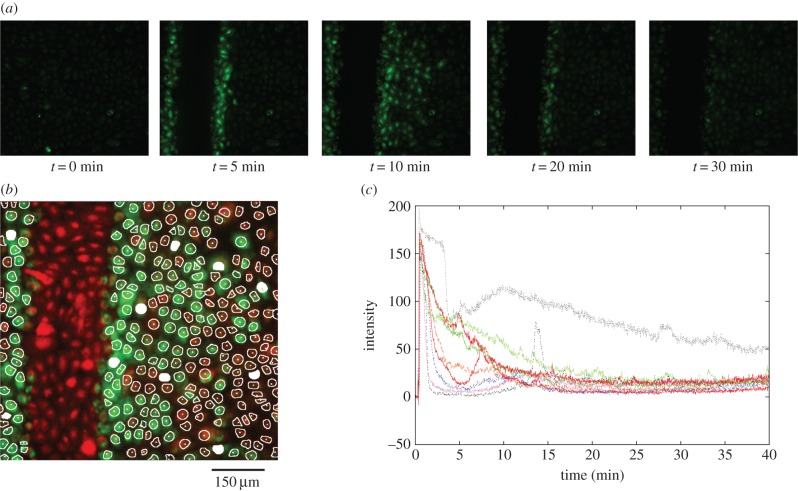
Calcium transients triggered by scratch wounding of the urothelial cell monolayer in a perfusion flow chamber. (*a*) Time-lapse images showing wounding and the subsequent activation of cells due to mechanical deformation of cells and the release of extracellular signalling factors around the wound site. Cells distal to the wound site show transient activation then return to baseline. Cells proximal to the wound site remain activated for an extended period following wounding. (*b*) The set of 253 identified ROIs. A strip of cells on the left side of the image has been deliberately excluded as these cells are removed during the wounding process. (*c*) Calcium transients for illustrative 10 cells identified by the ROI algorithm, indicated by the white-filled ROIs in *b*. As for the ATP experiment, considerable variety was observed in individual responses. Colours are arbitrary and for visual clarity.

A time-lapse sequence of fluorescence microscopy images illustrating the changes in cytosolic calcium concentration triggered by wounding is shown in figure 5*a*. As with the ATP experiment, it was immediately apparent that there was a significant amount of variation in the responses of individual cells in the population. Figure 5*b* shows the result of applying the segmentation algorithm to the combined red and green images. In this case, 253 ROIs (individual cells) were identified, representing around 98% of the total population (found by manual counting). Note that cells in the region where wounding occurred were excluded as these cells were either removed upon wounding or were too badly damaged to remain viable. Figure 5*c* shows the calcium transients of 10 cells, selected in order to illustrate the range of transients seen. A broad range of responses were apparent, including a variety of peaks and plateaus, although it is notable that cells in the wounding experiment did not exhibit any of the spiking or bursting behaviours seen upon exogenous application of ATP (above).

**Figure 6.**
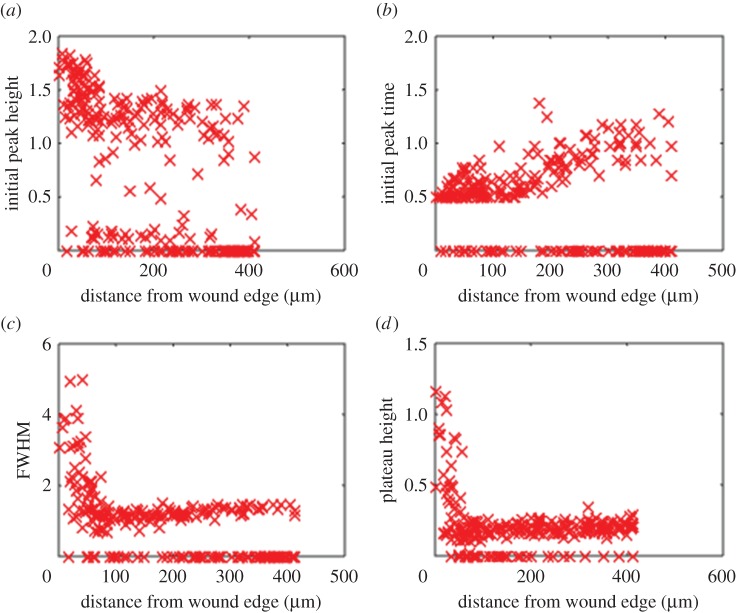
Spatial dependence of the calcium transients observed in the wounding experiment. (*a*) Cells closer to the wound have a tendency to produce slightly larger initial peaks (in arbitrary units), although this correlation is weak. (*b*) There is a weak trend for the peak response time (in minutes) to increase with distance from the wound edge. (*c*) A much stronger relationship with wound proximity can be observed in the FWHM (in minutes). A rapid increase in FWHM begins at around 100 μm from the wound edge, indicating that proximal cells tend to have a long, sustained plateau following the initial peak. (*d*) The same relationship can be observed in terms of the plateau height (in arbitrary units), indicating that proximal cells tend to have plateaus of much greater magnitude than distal cells.

An analysis was conducted in order to ascertain whether there was any relationship between the characteristics of the cytoplasmic calcium signatures recorded and the distance from the wound edge. In figure 6*a*, the initial peak height is plotted against the spatial distance of the cell from the wound edge. Cells exhibited a wide range of calcium transients, including those with large initial peaks and others with very small or no initial peak. However, it can be seen that cells within approximately 100 μm of the wound edge tended to have larger initial peaks than those located further away. In figure 6*b*, the time post-wounding of the initial peak is plotted as a function of distance from the wound edge. There is no obvious relationship between peak time and distance close to the wound edge, though at distances greater than approximately 150 µm, a trend for increasing peak time with increasing distance can be seen. Figure 6*c* illustrates the relationship between spatial distance from the wound site and the FWHM. Overall, the cells can be divided into two broad groups: those with a zero FWHM, indicating a transient with no initial peak, and those with a non-zero FWHM. Cells that showed an initial peak and were more than 100 μm from the wound edge all had very similar FHWMs of around 1 min, whereas cells closer to the wound tended to have a larger FWHM, reaching values as high as 5 min. Figure 6*d* illustrates the relationship between spatial distance and plateau height. In this case, the magnitude of the plateau, when present, increased sharply for cells closer than approximately 80 μm. These observations indicate that when a response is elicited, cells proximal to the wound edge are characterized by a large initial peak followed by a sustained elevation in cytosolic calcium, whereas distal cells are dominated by the large initial peak followed by a return close to baseline.

**Figure 7.**
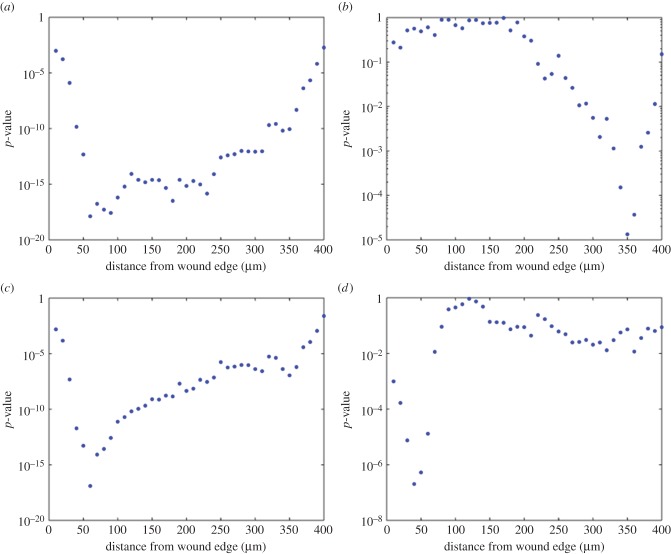
Statistical significance of the spatial dependence of the calcium transients observed in the wounding experiment. Cells were binned according to distance from the wound edge in 10 μm intervals. For each quantitative calcium signature characteristic, statistical significance was evaluated using a Mann–Whitney test for distance less than or equal to *x* compared with distance greater than *x* for 10 < *x* < 400 μm. (*a*) Initial peak height; (*b*) initial peak time; (*c*) FWHM and (*d*) plateau height.

In order to examine this effect in more detail, statistical significance tests were applied to examine relationships between calcium signature characteristics and distance from the wound edge. Calcium signatures were ‘binned’ at 10 μm intervals according to distance of the cell from the wound edge. For each characteristic recorded in figure 6, a two-tailed Mann–Whitney *U* test was used to test the hypothesis that signatures recorded less than distance *x* from the wound edge were significantly different from those located at greater than *x* from the wound edge, where 10 μm < *x* < 400 μm. The derived *p*-values for each characteristic are plotted on a logarithmic scale as a function of *x* in figure 7. It can be seen that initial peak height (figure 7*a*), FWHM (figure 7*c*) and plateau height (figure 7*d*) all have a statistically significant dependence on distance from the wound edge. This significance is greatest (as indicated by minimum *p*-value) at 60–100 μm for initial peak height, approximately 60 μm for FWHM and approximately 40 μm for plateau height. At distances greater than 100 μm, differences in plateau height are no longer statistically significant at the 99% confidence level. Thus, we select 100 μm as the nominal threshold to differentiate between what we henceforth refer to as ‘proximal’ (*x* < 100 μm) and ‘distal’ (*x* > 100 μm) cells. Table 4 summarizes the results of statistical analysis using the Mann–Whitney *U* test to compare features of the calcium signatures between proximal (less than 100 μm) and distal cells. Electronic supplementary material, table S1 contains the full analysis.

**Table 4.** Results of Mann–Whitney *U* test performed on extracted features of calcium transients of proximal (less than 100 μm from wound edge) and distal cells. * indicates statistically significant difference where *p* < 0.05; ** extremely significant difference (*p* < 0.001). Peak and plateau heights/intensities are in arbitrary units.

	proximal cells (*n* = 76)		distal cells (*n* = 178)		
transient feature	mean	s.d.	mean	s.d.	*p*-value
initial peak height	1.301	0.4895	0.5484	0.5854	<0.0001**
peak time (min)	1.033	3.275	0.5132	0.8725	0.5997
FWHM (s^−1^)	3.176	4.657	0.7623	0.9670	<0.0001**
plateau height	0.3156	0.2919	0.1648	0.08274	0.0290*

**Figure 8.**
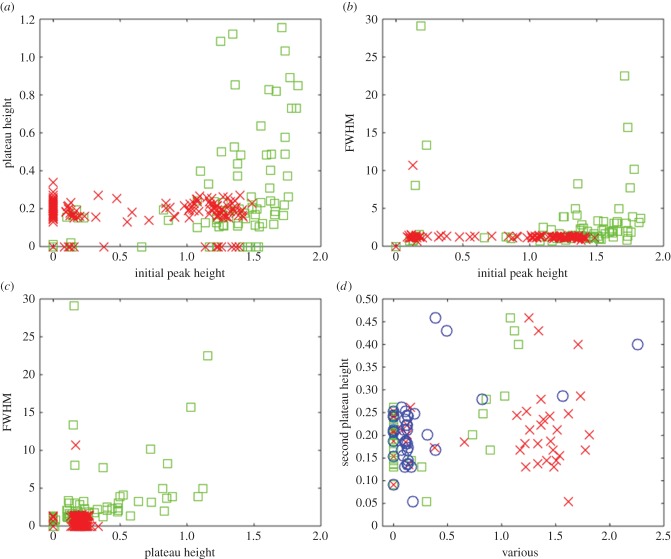
Detailed analysis of the calcium transients from the wounding experiment shown in figure 5*c*. We define cells within 100 μm of the wound site as proximal, and cells further away as distal. (*a*) Initial peak height versus plateau height (both in arbitrary units) for cells distal to the wound edge (red, ×) and cells proximal to the wound edge (green, □). (*b*) Initial peak height versus FWHM (in minutes) for the same population of cells. (*c*) Plateau height versus FWHM. (*d*) Initial peak height (red, ×), plateau height (green, □), and FWHM (blue, ○), (scaled by a factor of 1/10) versus second plateau height, for cells which exhibited two distinct plateaus.

Figure 8 shows four plots that are analogous to those used to examine the different classes of transient identified in the ATP experiment (as shown in figure 4). Figure 8*a* shows the plateau height as a function of the initial peak height. Cells proximal to the wound site predominantly fall into a single, diffuse cluster with a large initial peak and a wide range of plateau heights, whereas cells distal to the wound site include cells with a large initial peak (bottom right) and those with a smaller initial peak (bottom left). In both cases, the plateau which follows is of small amplitude.

Figure 8*b* shows the FWHM as a function of the initial peak height. The same clusters are preserved in this plot, which further indicates that cells distal to the wound site have very rapidly decaying initial peaks, falling to half maximum within 1–2 min, while those proximal to the wound site have initial peaks that may take much longer to decay. Figure 8*c* shows the FWHM as a function of plateau height, where the distal cells are grouped into a single large cluster, confirming the observation that these cells tend to have rapidly decaying initial peaks followed by a shallow plateau. The responses of the proximal cells are more widely dispersed, reflecting the greater spread of plateau heights and rates of initial peak decay shown in figure 8*a*,*b*. This indicates that there is very little correlation between the plateau height and the FWHM of the initial peak.

Cells in the wounding experiment did not exhibit spiking or bursting, which appears to require the sustained stimulation generated by prolonged exogenous application of ATP. Several cells, however, exhibited a complex, dual plateau response in which an initial, large plateau was followed by a transition to a second, lower plateau; a response type that was not observed in the ATP experiment. The signatures of these cells are examined in more detail in figure 8*d*, which reveals the initial peak height, first plateau height, and FWHM versus the height of the second plateau. The clusters observed in the other plots are preserved as groups of points along the horizontal axis. There is very little correlation between the initial peak height or the first plateau height and the second plateau height, indicated by the largely continuous distribution of second plateau heights extending up the vertical axis. Examination of the data suggests that cells exhibiting a longer decay time for the initial peak tended to generate a higher second plateau, whereas, as expected, cells that are unable to maintain the initial elevation in cytosolic calcium concentration following wounding did not exhibit a second sustained calcium elevation.

### 2.4. Collation of calcium transients

**Figure 9.**
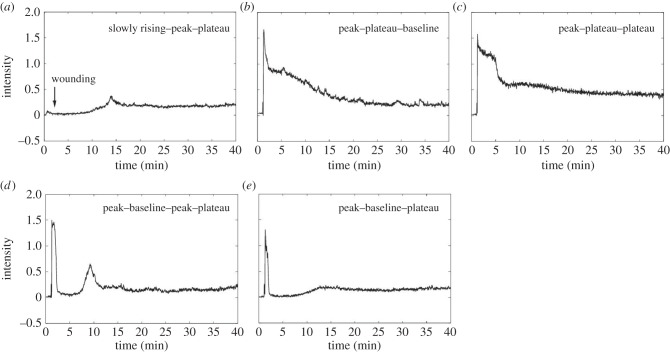
Examples of the five additional classes of calcium transient observed in the wounding experiment. (*a*) Slowly rising–peak–plateau, (*b*) peak–plateau–baseline, (*c*) peak–plateau–plateau, (*d*) peak–baseline–peak–plateau, and (*e*) peak–baseline–plateau. Intensities are in arbitrary units.

Examining the transients that make up the data points in figures 6 and 8, it can be seen that four of the transient types found in the ATP experiment were also found in the wounding experiment (from table 1, classes non-responding (1a), slowly rising (1b), peak–plateau (2a) and peak–plateau–peak–plateau (2b)). In addition, five new classes of transient were identified, as illustrated in figure 9*a–e* and listed in table 5. A common feature of these wound-specific transients is that they all eventually stabilize in a sustained, though small magnitude, plateau. The key differences in their shape is therefore found within the first 10 min following wounding, which contrasts with the observation made in the ATP experiment where the long-term behaviour was the dominant distinguishing feature, in spite of the applied ATP being washed off within 35 min.

**Table 5.** Classification of calcium transients evoked using the wounding protocol. Early response class is classified according to the response within the first 10 min post-wounding. Overall response class accounts for the behaviour over the subsequent 35 min. p, proximal cells (less than 100 μm) and d, distal (greater than 100 μm) from wound edge. Shaded cells indicate transient classes that were also identified in the ATP experiments (table 1).

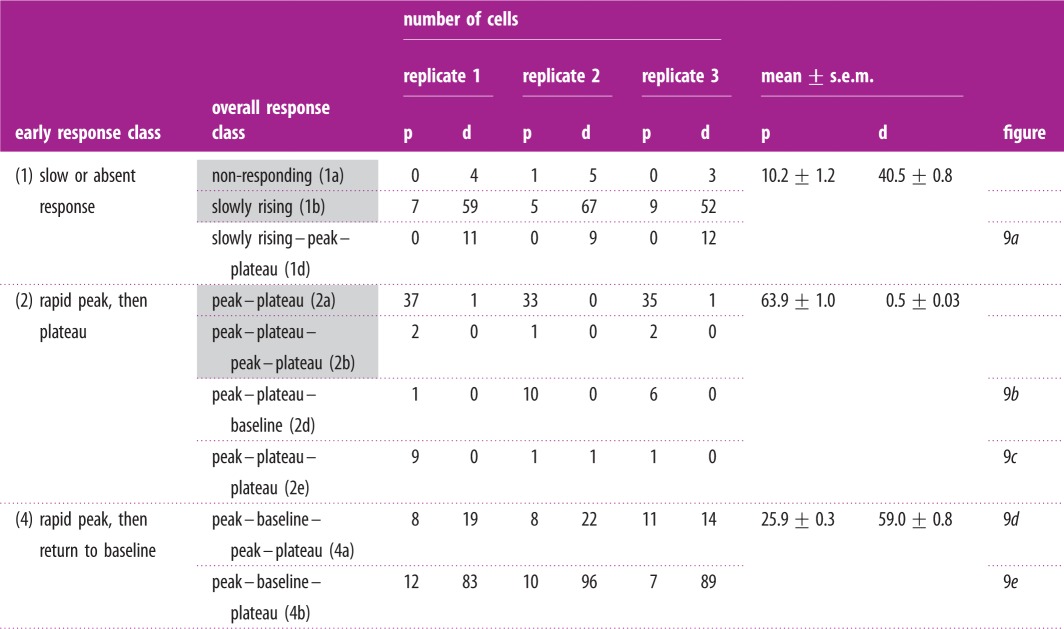

Table 5 shows a numerical breakdown of each of these five transient types, along with the four classes of transient also found in the ATP experiment. We have grouped the cells into proximal and distal populations (as discussed above) and subdivided the classes of transient based on their behaviour within the first 10 min into those exhibiting slow or absent responses, peak–plateau and peak–baseline groups. The number of cells assigned to each transient category from each of the replicates is shown in this table, and it can be seen that there is a close agreement between all three experiments.

The most striking feature observed from this table is the concentration of peak–plateau responses in the proximal cell population. This response accounts for less than 64% of this group, compared with less than 1% of the distal group. By contrast, distal cells have a much higher proportion of slowly responding cells (41% compared with 10%) and peak–baseline responses (59% compared with 26%). Cells proximal to the wound edge therefore typically exhibit a high amplitude initial peak, followed by a sustained elevation in cytosolic calcium. Cells distal from the wound edge do not generate the sustained plateau component of this response and instead tend to exhibit an initial peak followed by a return to baseline, or a gradual rise in cytosolic calcium that takes place over 10–15 min following wounding.

## 3. Discussion

In this paper, we have characterized in detail the variability in calcium transients observed in urothelial cells in response to application of an external agonist and to scratch wounding. Standard analysis of such data would typically involve the calculation of the mean response of the population, or by manual identification of around 10 individual cells (representing approximately 8% of the total population in a typical field of view), which would be designated as ROIs—a process that is time consuming and laborious. Each ROI is then used as a mask to examine the sequence of images, producing a time series of intensity for each cell that represents changes in cytosolic calcium concentration over time. Cells identified manually using the above process tend to be those that respond most strongly to the agonist, as these cells are easiest to delineate from the background, which strongly biases the results. Here, these issues were avoided by using an automated segmentation technique to delineate and extract individual calcium transients from over 95% of the visualized cells, thus eliminating any bias towards strongly responding cells. In order to analyse the large quantity of data this approach generated, an automated computer vision/image analysis algorithm that identifies and classifies the cell responses according to key features of the calcium transient was applied. Contrary to the picture of uniformity that may be incorrectly deduced from a mean-field approximation, or the relatively small numbers of cells examined in a conventional by-hand analysis, a wide range of response types within the urothelial cell population was observed.

We identified a total of 14 distinct transient types, which describes the entirety of responses recorded for urothelial cells after stimulation with exogenous ATP or scratch wounding. In our study, we identified two distinct sources of the variability that we have here termed *intrinsic*, which is due to natural variations from cell to cell (when cells are presented with an identical stimulus in the form of a soluble agonist), and *extrinsic* or context-specific, which arise due to external influences such as the spatial location of a cell relative to the wound site. In particular, we have shown that cells situated proximal (less than 100 μm) to a scratch wound have a significantly higher amplitude initial peak, of longer duration and a higher subsequent plateau, than cells further from the wound. As ATP release is implicated in epithelial wound repair [3,15], this observed difference in response could potentially arise as a consequence of direct or indirect mechanical stimulation of the proximal cells during scratch wounding.

There is a growing evidence in the cell biology/tissue engineering community that heterogeneity within biological tissues, including between cells derived from the same tissue, is an inherent property of the system which is critical for giving rise to emergent behaviours such as homeostasis [18] and plays a crucial role in the development and persistence of pathologies such as cancer (reviewed in [19]). Much of the experimental work conducted in our laboratory is focused on understanding tissue homeostasis and in particular, how heterogeneity in individual cell response can coordinate the emergent system-level behaviour within a cell population. Wound closure is an intrinsically population-orchestrated response and understanding both the extent and the origin of variability in a particular sub-population of cells is therefore of central importance—for instance, we have observations indicating that cells proximal to the wound edge exhibit an increased rate of migration during wound closure [3].

The automated techniques and algorithms that we have described to segment and extract individual cell data from a sequence of microscopy images, to analyse the extracted data and to classify the calcium transients obtained, are critical to achieving our goal of relating individual cell response to population behaviour. Our computational approach in analysing the experimental data permits a greater percentage of the cell population to be sampled and allows us to identify distinct sub-populations of response that would be missed using more conventional mean-field or by hand analysis. Neglecting certain groups of cells, for example, those that respond very little to the experimental stimuli used, or grouping the entire population together and extracting an average calcium transient would create a false picture of how individual cells respond and potentially lead us to overlook particular behavioural responses that may play an important role in the wound-closing response.

## 4. Material and methods

### 4.1. Experimental methods

Normal human urothelial cell cultures were established and maintained as described elsewhere [20]. For calcium imaging, NHU cells were seeded at a density of 5 × 10^4^ cells cm^−2^ onto collagen-coated glass coverslides in normal growth medium and incubated for 24 h before study. Cultures were washed in HEPES-buffered Hank's balanced salt solution (HH; 138 mM NaCl, 5 mM KCl, 0.3 mM KH_2_PO_4_, 4 mM NaHCO_3_, 0.3 mM NaHPO_4_, 1 mM MgCl_2_, 2 mM CaCl_2_ and 10 mM HEPES pH 7.4) and then loaded with 5 μM fluo4-AM and 5 μM fura red-AM for 30 min, as described previously [3,15]. The combination of fluo4 and fura red dyes was used as they, respectively, reduce and increase in intensity of fluorescence emission when bound to calcium, which permits ratiometric analysis and overcomes artefacts such as uneven dye uptake between cells. Cell cultures were rinsed in HH, before insertion of the coverslip into a laminar flow perfusion chamber (Warner Instruments, UK) and positioning on the stage of the Andor SD confocal microscope. HH buffer and agonists were perfused through the chamber using a computer-controlled automated perfusion pump system (PPS, Scientifica, UK) at a constant flow of 2 ml min^−1^ and images were captured at a minimum rate of 1 image s^−1^. Andor IQ software was used to determine the ratiometric change in intracellular calcium from individual cells and graphs were created using Microcal Origin software (OriginLabs). An example micrograph of a dye-loaded urothelial cell monolayer is shown in figure 1*a*. In ATP experiments, 25 μM ATP was washed over the monolayer for a fixed period of time. In wounding experiments, a strip of cells was removed by scratching with a fine glass pipette, which releases signalling factors from ruptured cells and mechanically stimulates cells near the wound site [3].

### 4.2. Cell segmentation and region of interest extraction

**Figure 10.**
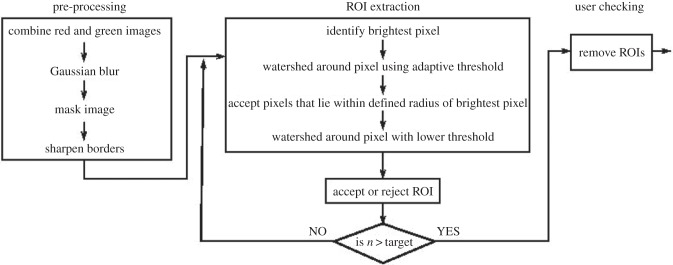
Schematic of the automated algorithm developed for ROI extraction from a sequence of immunofluorescence microscopy images.

The segmentation algorithm is a Matlab (Mathworks.com) script and composed of three phases: pre-processing, ROI extraction and user checking. During the pre-processing step, red and green frames are combined by summing the red and green channels then normalizing the result based on the minimum and maximum summed values. After smoothing using Gaussian blurring, the image is masked by setting all pixels below a specified threshold to zero. The mean brightness of a region around each pixel is calculated and pixels whose brightness is below a specified fraction of this mean brightness are set to zero; this creates clearer borders between cells by identifying pixels where the local intensity gradient is zero. In the ROI extraction step, the pixel with the highest intensity in the image is identified. The region around this pixel is selected using a watershed with an adaptive threshold under the constraint that each pixel is within a maximum radius of the initial pixel. These pixels become the ROI. A second watershed is then applied using a lower intensity threshold. These pixels become the ‘halo’ region and are set to zero, removing them from the image and helping to separate the cells. The ROI is rejected if it is too large or too small (according to user defined criteria set at the start of the process), in which case all pixels in the region are set to zero. This process is repeated until the number of ROIs specified by the user has been identified. In the user-checking step, the set of ROIs are displayed and individual ROIs can be selected and deleted by the user, as required. The remaining ROIs are then used as a mask to extract the temporal variation in intensity from the ratio of red and green channels for the full set of images. The time course of the average of the pixel values within each ROI at each time point is taken to provide a unique calcium signature for each cell. An outline of the automated algorithm for ROI extraction is shown in figure 10, and further information is given in electronic supplementary material, data 2.

### 4.3. Calcium signature metrics

For each calcium signature extracted using the Matlab code described above, a pre-processing step is applied that reduces the amount of noise in the trace. This involves taking a local average of points within regions where the calcium concentration is either slowly changing or not changing at all. This removes much of the noise in the slowly changing regions without discarding the information contained by the peaks and troughs in the rapidly changing parts. The cytosolic calcium concentration is then recorded at the maximum of the initial peak and during the plateau, along with the time taken for the concentration to fall to half that of the initial peak (the full-width half-maximum or FWHM). If the cell subsequently enters a spiking mode, the spiking rate is then also calculated by comparing the times of any subsequent spikes. From this information can be extracted an initial peak height and half-life, a plateau height and a spiking rate. An example of the classification algorithm is shown in figure 1*b*. These four features provide sufficient information to classify transients into several distinct groups, as described in the Results section.

## Supplementary Material

S1 Supplementary Tables

## Data accessibility

Immunofluoresence image sets collected and analysed as part of this study are available on DRYAD (http://doi.org/10.5061/dryad.mn8cs). The Matlab script used for the analysis of this data is available at http://staffwww.dcs.shef.ac.uk/people/D.Walker/research/research.html#CalSig.
